# The Propriety of Operative Interference in Tumors of the Breast

**Published:** 1893-06

**Authors:** Vard H. Hulen

**Affiliations:** Galveston, Texas


For Daniel's Texas Medical Journal.

### THE PROPRIETY OF OPERATIVE INTERFERENCE IN TUMORS OF THE BREAST.

BY VARD H. HULEN, M. D., GALVESTON, TEXAS.

[Read before Galveston County Medical Society, February 20, 1893]

TUMOR of the breast is a very common malady among women; and the disease is on the increase. According to Fordyce Barker, the mortality in New York from this cause was 400 to a million in 1875, while in 1885 it was 530 to the million. There are said to be over fourteen thousand patients dying every year in the United States from carcinoma alone.

Tumors are found in the breasts of both sexes, though the female breast is by far the most frequent site. The sex of the subject is not taken into consideration in the treatment.

In correctly classifying tumors there is considerable difficulty experienced; authorities differ more or less, yet all agree on the general terms, benign and malignant. The dividing line cannot be sharply drawn, but practically it is not of as great importance as it might at first seem.

Tubercular and syphilitic growths are here classed as benign tumors, though properly speaking, they are not tumors. These growths are to be treated on general principles. Cystic tumors, if few in number, only require evacuation, if many, excision of breast may be necessary.

Billroth says that as far as he knowns angioma and true neuroma do not occur in the breast.

Lymphatic tumors rarely require excision.

Lipoma, myxoma, enchondroma, and osteoma come under the general rules for treatment to be laid down later on. Adenoma and fibroma can be surely differentiated only under the microscope, but this is not necessary for correct treatment. These tumors should be excised as soon as detected; they may stimulate malignant growths, and it is a settled fact that fibromatous nodules may become carcinomas. It is best to remove them immediately on detection, for the critical point might be passed unnoticed if delay were practiced.

Occasionally after removal of these benign growths, secondary tumors of a cancerous nature appear. Paget thinks these are independent growths as a result of the previous local irritation. It must be remembered that all recurrent tumors are not cancers.

Billroth says "As a general rule, I might lay it down as a maxim that every continuously growing tumor should be extirpated of whatever nature it might be." There are any number of cases on record where tumors had every appearance for years of being benign, but finally assumed a cancerous nature.

Wyeth's advice is to excise, as soon as discovered, a tumor of the breast occurring in either sex after the 30th year of life.

Of the malignant tumors, there are two general classes. Sarcoma and carcinoma. Sarcomas are of several varieties, the spindle-celled greatly predominating; some authorities claim that not all sarcomas are malignant. Of carcinomas, there are two principal divisions; the encephaloid or soft cancer, and scirrhus or hard cancer. Gross states that nineteen-twentieths of all tumors of the breast are of the latter variety.

The only treatment for these tumors is early and complete removal by the knife of all diseased and *suspicious* tissues.

Dr. Geo. F. Shrady states that tumors which commence on the inner or stemal margin of the mamma are always, in his opinion, less promising than those upon the outer portions, due to the arrangements of the lymphatics of that region.

It is the recurrence of the disease that has to be contended with, for of malignant tumors, a vast majority do recur in a few months. Billroth is authority for the statement that if the diseased tissues are removed entirely the cancer will not recur,—though a *new* growth may form from the same (unknown) cause of the first tumor. It is certainly of vital importance that every particle of diseased tissue be recognized and removed, that it may be almost positively concluded that if a mammary gland contain even a small cancerous nodule it is wholly diseased, and if the nodule is found adherent in the least to the fascia of the pectoralis major, the muscle is diseased, and if a muscle is infected with cancer it seems to be diseased throughout its entire extent.

The closest attention must be given to the lymphatics, especially of the axilla, in all cases of suspected cancer. It is in detecting and entirely removing infected tissues that the skill and ability of the surgeon are shown, and not in amputating so beautifully the cancerous gland.

From the fact that almost all cancers recur sooner or later after the operation, a few surgeons do not advise surgical interference. Even should it be granted that a case of cancer was never *cured*, statistics certainly show that an operation will prolong life, and the patient be made more comfortable. It is satisfactory sometimes to only temporarily improve the conditition of the patient and soothe the mental distress, which accompanies this loathsome disease.

Weeden Cooke found that 409 out of 413 operated cases of cancer remained free from recurrence for six and a half months. Benjamin got an average of four and a half months in eighty cases. Von Winiwasten calculated from ninety-one cases that eleven remained free for over eleven months, and two out of the eleven were free one and a half years, two for two years, and one for three and a half years. Three years are taken as the standard time to represent permanent cures; Billroth takes the ground that after one year of complete absence of signs of recurrence, we should regard it a radical cure. He reports a number of cases that remained free for from thirteen months to twelve years. Volkman says "If a whole year passes after the operation without a local recurrence, glandular swelling or symptoms of internal affliction being demonstrated on most careful examination, we may begin to hope that a permanent good result has been obtained, of which we are usually certain after two years, and almost without exception, positive of after three years." On this basis he reports a number of cures.

Gross gives the percentage of recovery at about nine. Scirrhus may prove fatal in from six weeks to twenty years. The average duration according to Paget in unoperated cases is forty-three months, and operated cases fifty-seven and a half months, but these figures are undoubtedly too high. Sibley gives the duration of unoperated cases thirty-two months, and operated fifty-three months.

Gross gives the average life in the natural course of carcinoma as twenty-seven and a half months, when operated on as thirty-nine months. There is no doubt that life is prolonged for a few months at least, and there seems a chance of permanent cure in some few cases.

The cases of recurrence of carcinoma amount to about 75 per cent. of those collected in the basis of the three year limit. Dr. T. S. Dennis, of New York, in the May, '92, number of *Gaillard's Medical Journal*, states that with his cases, he has reducted the percentage of recurrence very much, He strongly advocates removing always without exception, the entire breast with the pectoral fascia and the lymphatic glands in the most insignificant cases of scirrhus. Kuster examined microscopically the lymphatic glands removed in 117 cases of carcinoma of the breast, and in only two of these 117 cases did he fail to find unmistakable evidence of carcinomatous infiltration.

To recapitulate: First. In case of a small tumor in the breast of a young patient that gives no indication of its presence other than it can be felt on examination and of slow growth would defer operating. If this same tumor was found in an older patient, would excise it at once, or if this tumor commenced to grow more rapidly, would then operate, or if there were a family history of cancer, would extirpate the tumor as soon as detected. If the presence of the growth caused the patient to be in constant dread of cancer, would remove it.

Second. If the tumor were large, and caused inconvenience, it should be excised.

Third. If the mammary gland were affected by the growth, that is, if the veins were enlarged, or the breast feverish or tender in touch, or if affected by lancinating pains at times, or the tumor were slightly adherent to the gland, would amputate the breast at once; and if the glands of the axilla were tender or enlarged, would dissect them out also.

Fourth. In case of early detection of cancer with an absence of metastatic growths, would amputate the breast and excise the glands of the axilla, in order to prolong life, and with a slight hope of a permanent cure.

Fifth. In hopeless cases would operate if requested to do so in order to relieve the pain and remove the distressing and disgusting growth and foul odor, provided we could expect the patient to survive the operation a reasonable length of time, the general condition and circumstances of the patient are always to be taken into consideration.

Sixth. Recurrent growths should be at once removed if no visceral complication be detected, for such removal certainly prolongs life, and lessens the risk of further infiltration or involvement of internal organs.

The propriety of an operation for tumor of the breast is not infrequently presenting itself to the surgeon, and a discussion of the subject will be interesting and instructive. It is to provoke this discussion that a paper so lacking in originality is presented to the society.

---

For Daniel's Texas Medical Journal:

